# Deciphering how *LIP2* and *POX2* promoters can optimally regulate recombinant protein production in the yeast *Yarrowia lipolytica*

**DOI:** 10.1186/s12934-016-0558-8

**Published:** 2016-09-20

**Authors:** Hosni Sassi, Frank Delvigne, Tambi Kar, Jean-Marc Nicaud, Anne-Marie Crutz-Le Coq, Sebastien Steels, Patrick Fickers

**Affiliations:** 1 Biotechnology and Bioprocesses, Université libre de Bruxelles, Avenue F.D. Roosevelt 50, 1050 Brussels, Belgium; 2 Microbial Processes and Interactions, University of Liège-Gembloux AgroBio Tech, Passage des Déportés, 2, B-5030 Gembloux, Belgium; 3 Micalis Institute, INRA, AgroParisTech, Université Paris-Saclay, 78350 Jouy-en Josas, France

**Keywords:** *Yarriwia lipolytica*, *POX2*, *LIP2*, Promoter regulation, Recombinant protein, Carbon source, Co-substrate

## Abstract

**Background:**

In recent years, the non-conventional model yeast species *Yarrowia lipolytica* has received much attention because it is a useful cell factory for producing recombinant proteins. In this species, expression vectors involving *LIP2* and *POX2* promoters have been developed and used successfully for protein production at yields similar to or even higher than those of other cell factories, such as *Pichia pastoris*. However, production processes involving these promoters can be difficult to manage, especially if carried out at large scales in fed-batch bioreactors, because they require hydrophobic inducers, such as oleic acid or methyl oleate. Thus, the challenge has become to reduce loads of hydrophobic substrates while simultaneously promoting recombinant protein production. One possible solution is to replace a portion of the inducer with a co-substrate that can serve as an alternative energy source. However, implementing such an approach would require detailed knowledge of how carbon sources impact promoter regulation, which is surprisingly still lacking for the *LIP2* and *POX2* promoters. This study’s aim was thus to better characterize promoter regulation and cell metabolism in *Y. lipolytica* cultures grown in media supplemented with different carbon sources.

**Results:**

p*POX2* induction could be detected when glucose or glycerol was used as sole carbon source, which meant these carbon source could not prevent promoter induction. In addition, when a mixture of glucose and oleic acid was used in complex medium, p*POX2* induction level was lower that that of p*LIP2*. In contrast, p*LIP2* induction was absent when glucose was present in the culture medium, which meant that cell growth could occur without any recombinant gene expression. When a 40/60 mixture of glucose and oleic acid (*w/w*) was used, a tenfold increase in promoter induction, as compared to when an oleic-acid-only medium was observed. It was also clear that individual cells were adapting metabolically to use both glucose and oleic acid. Indeed, no distinct subpopulations that specialized on glucose versus oleic acid were observed; such an outcome would have led to producer and non-producer phenotypes. In medium containing both glucose and oleic acid, cells tended to directly metabolize oleic acid instead of storing it in lipid bodies.

**Conclusions:**

This study found that p*LIP2* is a promoter of choice as compared to p*POX2* to drive gene expression for recombinant protein production by *Y. lipolytica* used as cell factory.

**Electronic supplementary material:**

The online version of this article (doi:10.1186/s12934-016-0558-8) contains supplementary material, which is available to authorized users.

## Background

Non-conventional model yeast species such as *Pichia pastoris*, *Hansuluna polymorpha*, and *Yarrowia lipolytica* have received much attention in recent years because they can serve as cell factories for producing heterologous proteins at lab or industrial scales [1, 2]. High yields of more than one hundred heterologous proteins have already been successfully obtained using *Y. lipolytica,* underscoring this yeast’s production potential [3]. When developing an efficient cell factory, the choice of the promoter driving recombinant gene expression is crucial. It therefore represents one of the key parameters to be optimized. At present, few promoters have been identified in *Y. lipolytica* and their regulation is not fully understood. Among them is the promoter derived from the *XPR2* gene, which encodes an alkaline extracellular protease. It was the first to be characterized and has been used to drive heterologous protein synthesis [4, 5]. However, full induction of this promoter requires high peptide concentrations and a pH above 6, conditions that are often unfeasible [6]. Constitutive promoters have also been considered, such as the one derived from the *TEF1* gene, which encodes translation elongation factor-1α [7], or the hybrid promoter hp4d, which is derived from the *LEU2* and *XPR2* genes [8, 9]. Although these constitutive promoters are highly efficient, they have a drawback: the high protein yields obtained from the early stages of culture may be detrimental to cell growth (e.g., the proteins produced are toxic to the host). Therefore, identifying regulated promoters that can be used to express recombinant genes in *Y*. *lipolytica* is a challenge.

*Yarrowia lipolytica* is known for its ability to assimilate hydrophobic substrates, such as methyl oleate [10, 11] and oleic acid [10, 12]. Consequently, promoters of the key genes involved in this metabolic process have been cloned and characterized. More specifically, they have been used to drive heterologous gene expression, as in the case of the promoter derived from the *POX2* gene that encodes acyl-CoA oxidase 2, which is involved in the first step of peroxisomal β-oxidation, and the promoter derived from the *LIP2* gene, which encodes the extracellular Lip2p lipase [9]. Using expression vectors based on p*LIP2*, greater quantities of enzymes such as Lip2p lipase have been produced in *Y. lipolytica* than in other cell factories such as *Pichia pastoris*. Using the *GAP* constitutive promoter, Wang and Coll (2012) obtained lipase activity levels of 13,500 U/mL using a glucose fed-batch process in a 10-L bioreactor [13]. In contrast, activity levels of 150,000 U/mL were obtained using a *LIP2* promoter and a tryptone-olive oil fed-batch process [11]. However, little else is known about these promoters’ regulation mechanisms. *POX2* promoter has been found to be induced in the presence of hydrophobic substrates such as fatty acids or triglycerides, and repressed or not induced in the presence of glucose or glycerol [14]. There is an additional layer of complexity: as highlighted elsewhere, the hexokinase Hxk1 is involved in the glucose-catabolite-mediated repression of the *LIP2* promoter [15]. Recently, Hussain and Coll (2015) reported on promoter engineering in *Y. lipolytica* [16]. Their findings highlight that promoter strength can be fine-tuned through the engeneering of the different promoter constitutive components.

This study examined how p*POX2* and p*LIP2* regulation was affected by medium type and, more interestingly, by carbon source. To this end, the promoters were fused with a reporter gene that codes for a red fluorescent probe that was used to quantify promoter induction either at the global or at the single cell level.

## Results

### Impact of carbon source on promoter regulation

**Fig. 1:**
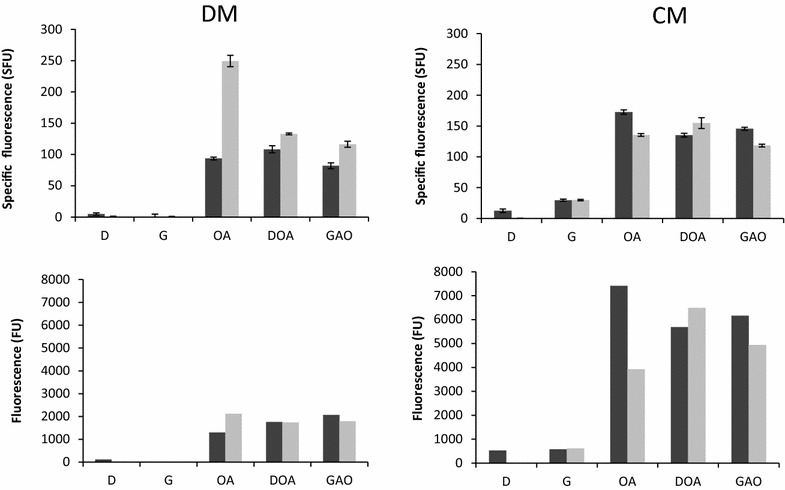
Comparison of p*LIP2* and p*POX2* induction using DsRed fluorescence as a proxy. Strains JMY2656 (p*POX2*-*RedStar2*; in *dark gray*) and JMY3742 (p*LIP2*-*RedStar2*; in *light gray*) were grown in culture media until carbon source depletion (i.e., 48 h). Complex and defined media (CM and DM, respectively) were used, to which the following carbon sources were added: *D* glucose; *G* glycerol; *OA* oleic acid; *DOA* glucose and oleic acid (ratio of 1 to 1 C-mol); and *GOA* glycerol and oleic acid (ratio of 1 to 1 C-mol). The values depicted are the mean raw fluorescence and specific fluorescence calculated from three independent replicates; the *bars* represent the standard deviation. *FU* fluorescence unit; *SFU* specific fluorescence unit

DsRed fluorescence was used to quantify p*POX2* and p*LIP2* induction levels in strains JMY2656 (p*POX2*-*RedStar2*) and JMY3742 (p*LIP2*-*RedStar2*) grown in defined medium (DM) and complex medium (CM) supplemented with various carbon sources. Specific fluorescence (i.e., biomass-corrected fluorescence) and raw fluorescence are depicted in Fig. 1. In defined medium supplemented with glucose (DMD) or glycerol (DMG), no fluorescence was detected for either strain, indicating that neither p*LIP2* nor p*POX2* had been induced. This result concurs with what has been seen in previous studies in which *LacZ* was used as a reporter [17]. In complex medium supplemented with glucose (CMD), p*LIP2* was not induced, while p*POX2* was slightly induced. Both were induced at low levels in complex medium supplemented with glycerol (CMG). In all media containing oleic acid (OA), specific fluorescence, and therefore induction levels, were significantly higher, especially for strain JMY3742 grown in DMOA. No significant differences in specific fluorescence were observed for strain JMY3742 grown in complex medium supplemented with glucose and oleic acid (CMDOA) or glycerol and oleic acid (CMGOA) versus in CMOA. This finding underscores that the repressive effects of glucose and glycerol are alleviated when oleic acid is also present. For JMY2656, there was a slight decrease in specific fluorescence in CMDOA (135 SFU) and CMGOA (154 SFU) versus in CMOA (172 SFU). JMY2656 showed similar specific fluorescence in DMDOA, DMGOA, and DMOA. In contrast, it was much reduced for JMY3742 grown in DMDOA (132 SFU) or DMGOA (116 SFU), as compared to when the strain was grown in DMOA (249 SFU). Raw fluorescence, a proxy for global induction levels, was much greater for strains grown in CM versus DM. This observation could be lined with the higher biomass yield at the end of the culture in CM medium compared to DM medium (28.7 ± 2.7 and 15.3 ± 2.8 OD 600 nm, respectively). Moreover, p*LIP2* induction was significantly greater in CMDOA and CMGOA than in CMOA. Cultures of strain JMY778 expressing the LacZ gene encoding β-galactosidase under the control of pLIP2 gave similar results (i.e., 117 ± 4 and 95 ± 5 Miller unit in CMOA and CMDOA, respectively). This finding means that with 50 % less oleic acid added to the culture medium, similar or higher induction levels could be obtained. Given these results, the impact of carbon sources on p*LIP2* regulation in cultures grown in CM was investigated in greater detail.

### *LIP2* promoter regulation and carbon uptake

**Fig. 2:**
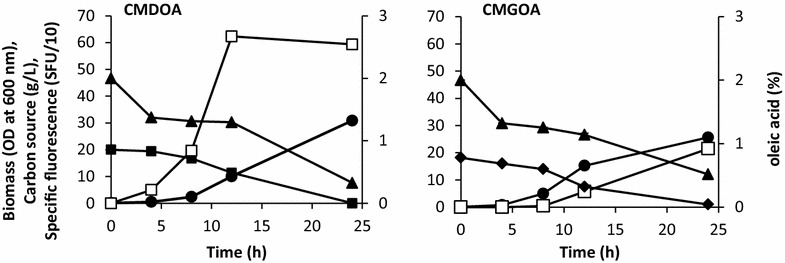
Accumulated biomass (●) and specific fluorescence (□) over time for cultures of strain JMY3742 (p*LIP2*-*RedStar2*) grown in CMDOA versus CMGOA. The symbols are the means calculated from three independent replicates. The standard deviations were less than 10 % of the mean values. The symbols are as follows: glucose, ■; oleic acid, ▲; and glycerol, ◆. *SFU* specific fluorescence unit. Media abbreviations are as in Fig. 1

To better understand p*LIP2* regulation, biomass production, carbon-source uptake, and DsRed fluorescence were characterized for JMY3742 cultures grown in CMD, CMG, CMOA, CMDOA, and CMGOA. Table 1 details JMY3742 growth and carbon uptake rates, and Fig. 2 depicts dynamics of cell growth, carbon source consumption and specific fluorescence for cultures performed in CMDOA and CMGOA. As can be seen, both carbon sources were consumed simultaneously from the beginning in CMDOA and CMGOA. This result suggests that *Y. lipolytica* is able to metabolize oleic acid and glucose or glycerol at the same time. However, the specific oleic acid uptake rate (r_OA_) was reduced by 20–30 % in CMDOA [i.e., 0.07 g/(gDCW h)] and CMGOA [i.e., 0.08 g/(gDCW h)], as compared to in CMOA [i.e., 0.10 g/(gDCW h)]. Similarly, the specific glucose uptake rate (r_D_) in CMDOA was 10 % less than in CMD [i.e., 0.09 and 0.10 g/(gDCW h), respectively]. In contrast, the specific glycerol uptake rate (r_G_) was twofold lower in CMGOA. For all conditions, the growth rate (r_x_) and the maximum biomass (X_max_) obtained at the end of the growth phase (i.e., after 48 h; growth: mean of 0.25 gDCW/(L h) and maximum: 8.53–9.12 gDCW/L) were in the same range. The only exception was for cultures grown in CMG, where mean growth was 0.15 gDCW/(L h) and maximum biomass was 4.4 gDCW/L.

**Table 1:** Growth and uptake dynamics of strain JMY3742 cultured in CMDOA, CMGOA, CMD and CMG

Culture medium	r_X_ gDCW/(L h)	r_D_ g/(gDCW h)	r_G_ g/(gDCW h)	r_OA_ g/(gDCW h)	r_luo_ FU/h	X_max_ gCDW/L
CMDOA	0.26	0.08	–	0.07	108.12	9.12
CMGOA	0.25	–	0.11	0.08	82.23	8.91
CMOA	0.26	–	–	0.11	68.61	8.90
CMD	0.26	0.10	–	–	ND	8.53
CMG	0.15	–	0.21	–	8.51	4.44

The values provided are the means of three independent replicates; the standard deviations were less than 10 % of the means

*r*_*X*_ cell growth rate, *r*_*fluo*_ specific induction rate, *r*_*G*_ specific glycerol uptake rate, *r*_*D*_ specific glucose uptake rate, *r*_*OA*_ specific oleic acid uptake rate, *X*_*max*_ final biomass. *ND* not detected

**Fig. 3:**
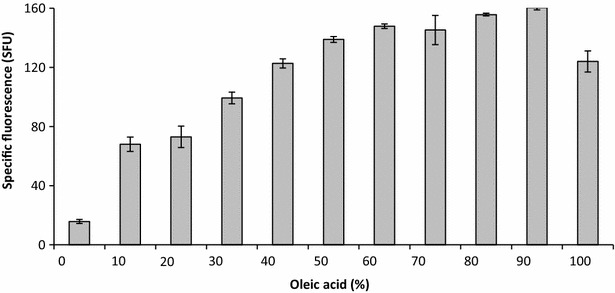
Specific fluorescence after 60 h for cultures of strain JMY3742 grown in CMDOA (see Fig. 1 for definition) containing different ratios of glucose to oleic acid. The data depicted are the means calculated from three independent replicates. *SFU* specific fluorescence unit

DsRed fluorescence and, therefore p*LIP2* induction, was 57 % (6482 SFU) and 24 % (4936 SFU) higher in CMDOA and CMGOA, respectively, than in CMOA (3926 SFU) (data not shown). Moreover, the specific induction rate (r_fluo_) was 37 % higher in CMDOA than in CMOA. This finding highlights that p*LIP2* induction was significantly greater when strain JMY3742 was grown in a medium containing a mixture of glucose and oleic acid (0.5/0.5 C-mol). To further explore the potential boost provided to p*LIP2* induction by growth in CMDOA, strain JMY3742 was grown for 60 h in different CMDOAs, which varied in their ratios of glucose to oleic acid (total carbon concentration of 1.8 Cmol/L); specific fluorescence was then determined. The results show that p*LIP2* induction varied depending on the glucose to oleic acid ratio (Fig. 3). It increased almost linearly when oleic acid levels ranged from 0 to 0.5 C-mol. It then plateaued between 0.6 and 0.9 C-mol. Finally, when oleic acid was the sole carbon source, p*LIP2* induction declined by 23 %. Under all the experimental conditions tested, similar final biomass values were obtained (data not shown). The results of this experiment highlight that mixtures of glucose and oleic acid, in which the amount of glucose ranges from 0.1 to 0.4 C-mol, can increase p*LIP2* induction by almost 20 %.

### Transcriptional analysis

**Fig. 4:**
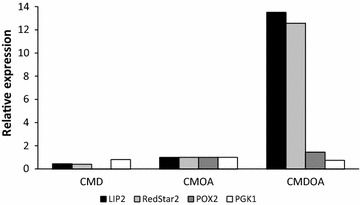
Transcription of mRNA from *LIP2* (encodes an extracellular lipase), *RedStar2* (encodes the DsRed fluorescent protein), *POX2* (encodes acyl-CoA oxidase 2), and *PGK1* (encodes 3-phoshoglycerate kinase) in strain JMY3742 after 24 h of growth in CMD, CMOA, and CMDOA. The data were processed using the Livak method, and expression levels were normalized based on the results obtained in CMOA. The values are the means calculated from three independent replicates, and the standard deviations were less than 10 % of the means. Media abbreviations are as in Fig. 1

To confirm that the *LIP2* promoter was being induced at higher levels in *Y. lipolytica* grown in CMDOA, RT-qPCR was used to quantify the transcription of the *RedStar2* and *LIP2* genes. These two genes were targeted since their expression was controlled by the same promoter in strain JMY3742. To facilitate comparisons, mRNA levels were normalized to those obtained for cultures grown in CMOA. The results show that *RedStar2* and *LIP2* were transcribed relatively less in CMD, although transcription levels increased 13.5- and 12.5-fold, respectively, in CMDOA (Fig. 4). This finding once again confirms that using a mixture of glucose and oleic acid significantly increases p*LIP2* induction.

The transcription levels of two key genes involved in glucose and oleic acid metabolism were also quantified. *POX2* encodes the acyl-CoA oxidase 2 involved in fatty-acid catabolism [15], and *PGK1* encodes the phosphoglycerate kinase that catalyzes the conversion of 1.3 bisphosphoglycerate into 3-phosphoglycerate during glycolysis [18]. Transcription levels were once again normalized to those obtained for cultures grown in CMOA. The expression of *PGK1* was relatively lower in CMD and CMDOA (20 and 25 %, respectively; Fig. 4). This finding concurs with that of a previous study, in which *PGK1* expression was found to be greater when a non-glycolytic substrate was used [18]. In contrast, *POX2* expression is modulated by the medium composition. For instance, transcription declined ten-fold in CMD but increased 46 % in CMDOA. This result highlights that the β-oxidation pathway is more active in the presence of both glucose and oleic acid.

### Analysis of carbon-source uptake by individual cells

**Fig. 5:**
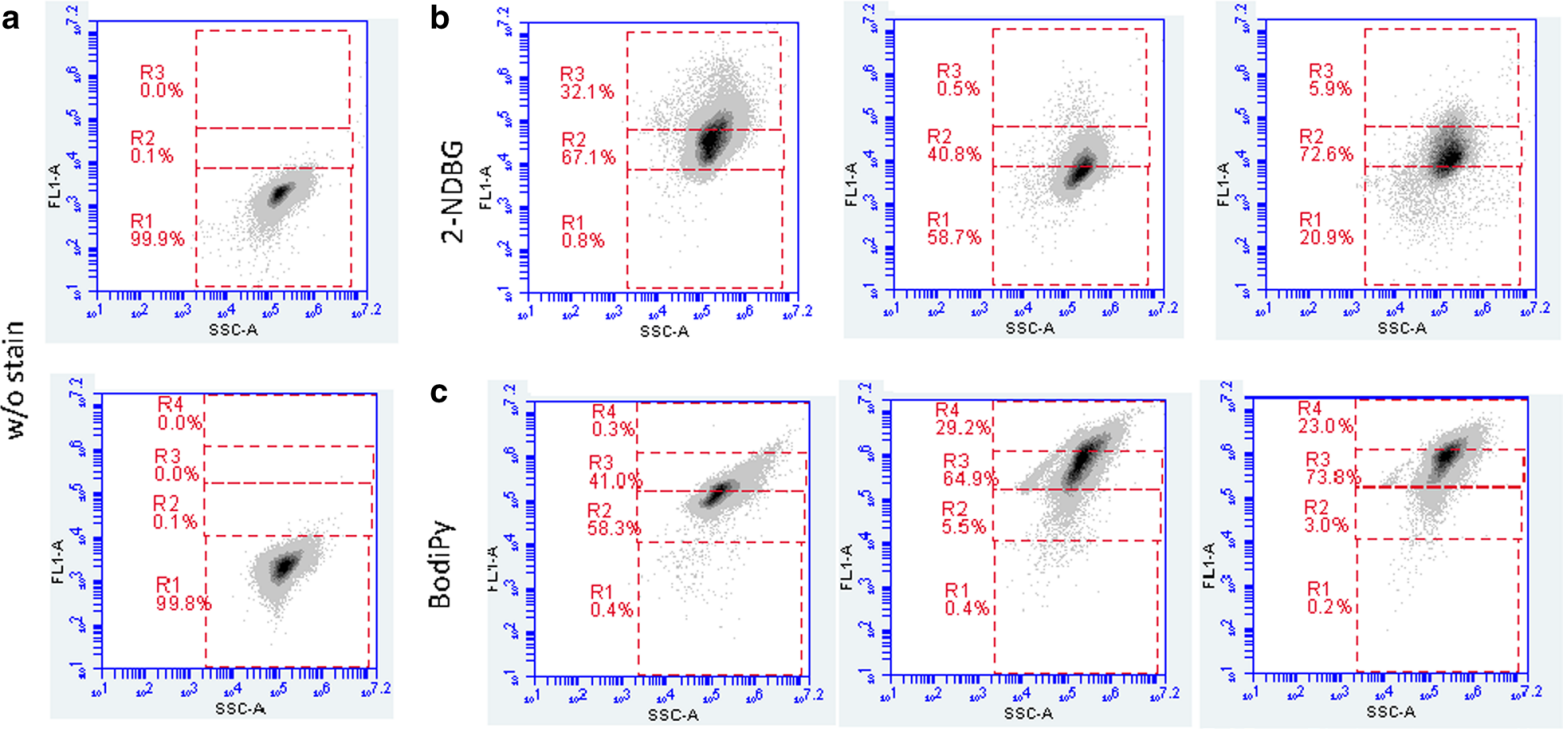
Glucose transport activity and intracellular lipid accumulation in individual cells in JMY3742 cultures grown in CMD, CMOA, and CMDOA. At the mid-log phase, cells were harvested, washed, and resuspended in a solution containing 2-NDBG (**b**) or Bodipy (**c**) to characterize glucose transport and fatty-acid accumulation, respectively. Levels of green fluorescence in 40,000 cells were analyzed after a 20-min incubation period using the FL1-A channel of a flow cytometer. Control strains without any straining are shown in **a**. Gates R1 to R4 are described in the “Methods” section. Media abbreviations are as in Fig. 1

As shown in Fig. 2, *Y. lipolytica* seems to be able to co-consume glucose and oleic acid. To further characterize this phenomenon at the single-cell level, glucose uptake capacity was quantified using the fluorescent glucose analog 2-NDBG [19], which cannot be metabolized. Flow cytometry was utilized to measure intracellular 2-NDBG fluorescence, which served as a proxy for the glucose transport capacity of individual cells. In Fig. 5, the area delimited by gate 1 corresponds to the fluorescence signal recorded in the absence of 2-NDBG—the phenotype expected for a cell exhibiting no glucose uptake (WGU). Cells grown in CMD showed the greatest 2-NDBG uptake capacity, and thus glucose transport (mean fluorescence: 60.5 × 10^3^ FU; Table 2). The area delimited by gate 3 corresponds to the high-glucose-uptake phenotype (HGU). Cells grown in CMOA had a low glucose-uptake capacity (mean fluorescence: 8.5 × 10^3^ FU); 58.7 % of cells displayed the WGU phenotype. In CMDOA, an intermediate situation was observed (mean fluorescence: 22.8 × 10^3^ FU): 5.9 % of cells displayed the HGU phenotype, 20.9 % displayed the WGU phenotype, and 72 % displayed an intermediate phenotype. The results of this experiment suggest that heterogeneity within the cell population is the product of phenotype diversity as opposed to the presence of two distinct subpopulations, one that metabolizes only glucose and the other that metabolizes only oleic acid.

**Table 2:** Mean fluorescence of cells (in 1 × 10^3^ FU) after 2-NDBG uptake or Bodipy staining

Probe	Culture medium			
	Control	CMD	CMOA	CMDOA
2-NDBG	1.8	60.5	8.5	22.9
Bodipy	3.4	160	900	840

In a related experiment, the intracellular accumulation of lipids in lipid bodies was monitored using Bodipy. In Fig. 5, the area delimited by gate R2 corresponds to the low-lipid-accumulation phenotype (LLA), while gate R4 corresponds to the high-lipid-accumulation phenotype (HLA). Gate R3, which lies between R2 and R4, corresponds to an intermediate phenotype (ILA). In the different media tested, cells accumulated lipids with at least LLA or ILA phenotype. Not surprisingly, mean fluorescence was lowest in CMD (160 × 10^3^ FU) and highest in CMOA (900 × 10^3^ FU) (Table 2). In CMDOA, lipid accumulation was intermediate (mean fluorescence: 840 × 10^3^ FU, 73.8 % of the cell population with R2 phenotype); the lower levels of lipid accumulation imply that more oleic acid was available for direct catabolism. This assessment is supported by the slightly greater biomass produced in CMDOA (Table 1).

## Discussion

Promoters of the *LIP2* and *POX2* genes have been successfully used in the development of regulated expression vectors [20]. However, cell factories exploiting these promoters are difficult to manage, especially when large fed-batch bioreactors are used because low-water soluble inducers such as oleic acid or methyl oleate are required [11]. Thus, it would be extremely helpful to reduce the loads of hydrophobic substrates without affecting heterologous protein productivity. One strategy may be to replace a certain portion of the substrate (e.g., oleic acid) with a non-repressive hydrophilic co-substrate that can serve as an alternative carbon source. We used such a strategy in *Pichia pastoris* to reduce methanol loads [21, 22].

To date, however, no study has examined *POX2* and *LIP2* regulation in media containing a second carbon source in addition to oleic acid. Here, the objective was therefore to better characterize the regulation of p*POX2* and p*LIP2* when glucose or glycerol was employed as a co-substrate. Promoter regulation was investigated using DsRed, the fluorescent protein encoded by the *RedStar2* gene. This system was preferred over others, such as the LacZ/β-galactosidase system, because DsRed has high intracellular stability. Hence, its accumulation in cells over time reflects total induction during the culturing period. Moreover, based on the comparison of DsRed fluorescence and *RedStar2* mRNA expression, it appears that fluorescence is a good proxy for promoter induction, at least under the experimental conditions tested here.

In this study, two types of media were utilized: a defined medium derived from that of Gasmi et al. [23] and a complex medium developed by the authors [23]. Although both medium types yielded similar specific fluorescence levels, which meant that levels of promoter induction were similar, the results suggest that complex medium is to be preferred over defined medium because the latter yielded significantly less biomass. In complex medium supplemented with glucose, p*LIP2* production was completely repressed. This matches what was previously seen when a different reporting system was used [11, 17]. In media supplemented with glycerol, both promoters were slightly induced, suggesting that this carbon source is less suitable for producing biomass. Moreover, less biomass was obtained in complex medium supplemented with glycerol. Given these results, the study’s focus shifted to using glucose as a co-substrate.

In a previous study, it was found that oleic acid is a good inducer for both p*LIP2* and p*POX2* and that induction is medium dependent [24]. Here, similarly, p*LIP2* induction was greater than p*POX2* induction in defined medium supplemented with oleic acid; in the equivalent complex medium, the opposite was true. Based on the fluorescence results, p*LIP2* induction appeared to be greater in complex medium containing both oleic acid and glucose than in oleic-acid-only medium. To confirm these results, mRNA transcription levels for the native *LIP2* gene and the recombinant *RedStar2* gene, both of which are regulated by p*LIP2*, were quantified for strain JMY3742 grown in the presence of different carbon sources. In medium supplemented with both glucose and oleic acid, *LIP2* and *RedStar2* expression were tenfold higher than in oleic-acid-only medium. Our findings suggest that, when glucose is also present in the culture medium, more oleic acid is available for induction because part of the energy used in cell metabolism is furnished by the co-substrate. Using a simplified metabolic flux analysis, support for a similar hypothesis was found in *P. pastoris*; in that case, the promoter was methanol induced and sorbitol was used as a co-substrate [22]. These results also imply that *Y. lipolytica* is able to simultaneously metabolize oleic acid and a co-substrate such as glucose or glycerol. Glucose and glycerol co-consumption has recently been observed in *Y. lipolytica* strain IBT 446 [25]. In that study, the co-consumption of carbon sources was attributed to the absence of carbon catabolite repression. Indeed, the authors failed to identify homologs for relevant genes during their BLAST search. This finding stands in contrast to results obtained with other eukaryotic cell factories, such as *Saccharomyces cerevisiae*, that exhibit carbon catabolite repression in the presence of glucose [26]. Here, it has been clearly demonstrated that *Y. lipolytica* is able to simultaneously consume oleic acid and a co-substrate such glucose or glycerol.

Surprisingly, *POX2* was expressed at significantly higher levels (46 %) in medium supplemented with glucose and oleic acid than in medium containing just oleic acid, which suggests that β-oxidation is more active in the former. However, while the specific oleic acid uptake rate, r_OA_, was similar in both media, lipid accumulation in lipid bodies was somewhat lower in the glucose-and-oleic-acid medium. Indeed, the percentage of cells displaying the high-lipid-accumulation phenotype was smaller in the latter medium than in the oleic-acid-only medium. Therefore, when a co-substrate is available, cells seem prone to directly metabolize oleic acid instead of storing it in lipid bodies.

Furthermore, *PGK1* expression was slightly lower in the glucose-and-oleic-acid medium than in the oleic-acid-only medium. Since *PGK1* encodes a glycolytic enzyme, its lower levels of expression suggest a lower level of glucose catabolism in that medium. This observation fits with the lower glucose uptake rate, r_D_, observed in the glucose-and-oleic-acid medium and with the results of the 2-NDBG uptake experiments.

This study clearly shows that *Y. lipolytica* can simultaneously metabolize oleic acid and glucose. Nonetheless, the question arose: can all cells co-consume both carbon sources or are cultures composed of two subpopulations, each exploiting only one of the two carbon sources? The experiments carried out at the single-cell level suggest that individual cells can indeed co-consume both carbon sources.

## Conclusions

This study found that p*LIP2* should be a promoter of choice for the synthesis of recombinant proteins, especially on complex medium. Its failure to be induced in medium in which glucose was the sole carbon source allows *Y. lipolytica* cells to grow without any leaks of p*LIP2*-driven gene expression. Compared to the *POX2* promoter, the *LIP2* promoter also permits to use less oleic acid when glucose is available as co-substrate; the ideal glucose/oleic acid ratio appears to be 40/60. Moreover, the results of the flow cytometry experiments clearly demonstrate that individual cells can adapt to metabolize both glucose and oleic acid.

## Methods

### Plasmids, strains, media, and culture conditions

The plasmids, oligonucleotides, *Escherichia coli* strains, and *Y. lipolytica* strains used in this study are listed in Table 3. The *E. coli* strain Mach1T1 (Thermo Fisher Scientific, Belgium) was used in the transformation and amplification of the recombinant plasmid DNA. Bacteria were grown at 37 °C in Luria–Bertani medium supplemented with kanamycin sulphate as necessary (50 mg/L, Sigma-Aldrich, Belgium). The *Y. lipolytica* strains were grown at 28 °C in YPD or YNB supplemented to meet the requirements of auxotrophs [27]. To select the Ura^+^ clones, transformants were plated on YNBura (YNB containing 0.01 % uracil; Sigma-Aldrich). The defined medium (DM), which was derived from GNY medium [28], was composed of 0.93 g/L CaSO_4_·2H2O, 18.2 g/L K_2_SO_4_, 7.28 g/L MgSO_4_·7H_2_O, 4.4 g/L KOH, 26.7 mL/L H_3_PO_4_ 85 %, 10 mg/L FeCl_3_, 1 g/L glutamate, 5 mL/L of PTM1 solution (6 g/L CuSO_4_·5H_2_O, 0.08 g/L KI, 3 g/L MnSO_4_·2H_2_O, 0.2 g/L Na_2_MoO_4_·2H_2_O, 0.02 g/L H_3_BO_3_, 0.5 g/L CoCl_2_·6H_2_O, 20 g/L ZnCl_2_, 6.5 g/L FeSO_4_·7H_2_O and 5 mL/L H_2_SO_4_) as well as 2 mL/L of a vitamin solution (8 μg/L biotin, 200 μg/L thiamin, 4 μg/L myo-inositol). The complex medium (CM) was composed of 10 g/L yeast extract and 20 g/L tryptone. Both media contained a sodium phosphate buffer (50 mM; pH 6.8) and were supplemented with 1.8 C-mol/L of various carbon sources as follows: glucose (D), glycerol (G), oleic acid (OA), glucose and oleic acid (DOA; 0.9 C-mol each), or glycerol and oleic acid (GOA; 0.9 C-mol each)(see Additional file 1). A fatty acid stock solution (20 % oleic acid, 0.5 % Tween 20) was subject to sonication for 1 min to carry out emulsification; a Sonics Vibra-Cell™ Processor (USA) was used. Precultures were grown in 50-mL flasks containing 15 mL of YPD medium; they were incubated at 28 °C and kept at 250 rpm for 20 h. Culturing was performed under the same conditions but using 24-square deepwell microtiter plates (System Duetz, Enzyscreen, [29]. Culture volume was 2 mL, and inoculation was such that the initial optical density (OD) at 600 nm was 0.1. Cultures were performed in triplicate.

**Table 3:** Strains, plasmids, and primers used in this study

Yeast strains	Genotype	Source
W29	Wild-type strain	[30]
JMY2033	W29 *ura3*::*LEU2*ex-zeta, *leu2*-*270*, *xpr2*-*322*	[39]
JMY2656	JMY2033 p*POX2*-*RedStar2*	This study
JMY3742	JMY2033 p*LIP2*-*RedStar2*	This study

*E. coli* strain	Plasmid description	
JME509	JMP114	[35]
JME803	JMP62	[40]
JME1394	JMP62-type *LEU2*ex-p*TEF*-*RedStar2*	[41]
JME1491	JMP62-type *URA3*ex-p*TEF*-*RedStar2*	This study
JME1500	JMP62-type *URA3*ex-p*POX2b*-*RedStar2*	This study
JME1945	JMP62-type *URA3*ex-p*Lip2*-*RedStar2*	This study

Primers	Sequence (5′–3′)	Restriction site
LAM51	ACT*TTCGAA*ATGATAACGTTGGAGAAGAG	*BstB*I
LAM52	TC*GGATCC*TGAGGAGAGCTGGTACTTGG	*BamH*I
Lip2Qfo	CGAGGAACCCACTCTCTGG	
Lip2Qrev	GCTCAATCACAGAGTCGAGC	
RedStarQfo	CGAAGGTGAAGGTGAGGGTAG	
RedStarQrev	CCACTTGAAACCTTCTGGGAAG	
ACT-R	GGCCAGCCATATCGAGTCGCA	
ACT-F	TCCAGGCCGTCCTCTCCC	
POX2fo	GCCTGCATTCGACGACAGTTCTC	
POX2rev	CGTCGGAAACAACCTCATTGAGGC	
PGKfo	CAGGTGAGTACCCACGAAACGC	
PGKrev	GCATCCCAATTTGCCTTAGCATTTG	

### General genetic techniques

The media and techniques used for *Y. lipolytica* have been described elsewhere [30], and standard media and techniques were used for *E. coli* [31]. Restriction enzymes, DNA polymerases, and ligases were used in accordance with the manufacturer’s recommendations. Competent *E. coli* cells were obtained by the rubidium chloride method adapted from [32]. Genomic DNA was extracted from yeast cells as described elsewhere [33]. Polymerase chain reactions (PCRs) were performed with the primers mentioned in the text. Pyrobest DNA polymerase (Ozyme) was used for cloning, and ExTaq (Takara, France) was used for verifying genomic structures. DNA fragments were purified from PCR products using a QIAquick PCR Purification Kit (Qiagen, Germany) or recovered from agarose gels using the QIAquick Gel Extraction Kit (Qiagen). DNA sequencing was performed by GATC Biotech. Primers were designed with Primer 3 software (http://simgene.com/Primer3) and synthetized by Eurogentec (Seraing, Belgium).

### Construction of strains JMY2656 and JMY3742

p*LIP2* and p*POX2* were fused with the *RedStar2* gene, which codes for a fast-maturing variant of the red fluorescent protein DsRed [34]. They were then inserted into the zeta-docking platform of *Y. lipolytica* strain JMY2033. First, the *LEU2*ex selectable marker of plasmid JME1394 was exchanged with the *URA*ex marker of JMP114 using I-*Sce*I digestion-ligation; this process yielded JME1491. Second, a 1.6-kb fragment of the *LIP2* promoter [35] was amplified via PCR from W29 genomic DNA and flanked by *BstB*I and *BamH*I sites using the primer pair LAM51/LAM52. The *POX2* promoter was rescued from JMP62 as a 1-kb *Cla*I-*BamH*I fragment. The two promoters were cloned into JMP1491 at the *Cla*I-*BamH*I restriction sites. The resulting plasmids were *Not*I digested, and the promoter-*RedStar2* expression cassettes were purified on gels before being introduced into the zeta-docking platform of the *Y. lipolytica* strain JMY2033, which yielded strains JMY2656 and JMY3742.

### Analytical methods

Cell growth was estimated using either OD at 600 nm or dry cell weight (DCW), as previously described [21]. The concentration of glucose in the culture broth was determined using a BioMerieux RTU7500 Kit (BioMerieux, France). Oleic acid and glycerol concentrations were determined by HPLC on a HP Agilent 1100 series apparatus (Agilent Technologies, Belgium) using an Aminex HPX-87H ion-exclusion column (300 × 7.8 mm Bio-Rad). Glycerol was eluted with 5 mM H_2_SO_4_ at a flow rate of 0.5 mL/min at 30 °C, and its quantified using a refractive index detector. Oleic acid was eluted with a mixture of acetonitrile, H_2_O, and acetic acid (98:2:0.5; *v/v*) at a flow rate of 0.5 mL and was detected at 205 nm. Total nitrogen content was estimated via Kjeldahl’s method. A Kjeltec™ System I with a Kjeltec 1026 Distilling Unit and a 6120 Kjeldahl digestion system (Tecator AB, Sweden) was used. DsRed fluorescence was measured using black microplates (Microfluor™) and a FLUOstar/POLARstar Galaxy Microplate Reader (BMG Labtech, Germany); the excitation and emission settings were 510 and 590 nm, respectively. β-glactosidase was measured as described previously (Fickers et al. [17]).

### Transcriptional analysis

Samples of yeast cells (1 mL) were centrifuged at 5000×*g* for 5 min; they were then stored at −80 °C until further analyses could be performed to prevent RNA degradation. RNA extraction and DNAse treatment were carried out using a NucleoSpin RNA Kit (Macherey–Nagel, Germany) in accordance with the manufacturer’s instructions. Total RNA was quantified using spectrophotometry; a Thermo Scientific NanoDrop 2000 UV–Vis Spectrophotometer was employed. RNA quality was evaluated via agarose gel electrophoresis. Reverse transcription was performed in a total volume of 10 μL, which included 50 ng of total RNA using the Core Kit (Eurogentec, Belgium) in accordance with the manufacturer’s instructions. The target genes *LIP2*, *RedStar2*, *POX2*, and *PGK* were amplified using the primer pairs Lip2Qfo/Lip2Qrev, RedStarQfo/RedStarQrev, Pox2fo/Pox2rev, and PGKfo/PGKrev, respectively. The reference gene (ACT-encoding actin) was amplified using the ACT-F/ACT-R primer pair [36]. Average amplicon size was 200 bp. Quantitative amplification was carried out using a StepOne™ Real-Time PCR System (Applied Biosystems). The following reaction protocol was employed: 95 °C for 10 min, 40 cycles at 95 °C for 15 s, 60 °C for 1 min, and 95 °C for 15 s. Reaction mixtures were prepared using the Mesa GRreen qPCR MasterMix Plus Kit (Eurogentec) as follows: 12.5 μL of the Mesa Green qPCR Master Mix Plus solution was combined with 1 μL of each primer (0.2 μM), 5 μL of cDNA (5 ng), and 5.5 μL of DNase/RNase free water. Primer specificity was validated using a melting-curve analysis. The amplification efficiency of each oligonucleotide was similar (84–107 %). The Livak method was used to analyze the raw data, and the results were standardized using the data from the complex medium supplemented with oleic acid (CMOA) as a reference.

### Quantification of 2-NBDG uptake

The uptake of (2-(*N*-(7-nitrobenz-2-oxa-1,3-diazol-4-yl)amino)-2-deoxyglucose), or 2-NBDG, by *Y. lipolytica* cells was estimated as described elsewhere [37]. At the mid-log phase, 1 mL of culture broth was centrifuged at 3200*g* for 4 min at 4 °C. The cells were then washed with phosphate-buffered saline (PBS; pH 7.2) containing 5 % ethanol to remove any remaining extracellular oleic acid; they were then resuspended in fresh medium without a carbon source such that OD at 600 nm was 0.5. A 100-µL sample of this cell suspension was centrifuged. The resulting cell pellet was immediately placed on ice for a few seconds until 2-NBDG uptake was initiated by adding 20 µL of 2-NBDG (10 mM). After a 20-min incubation phase at 30 °C, the reaction was stopped by adding 100 µL of 10 % formalin. Then, 1 mL of PBS was added and the sample was analyzed using a BD Accuri™ C6 Flow Cytometer (BD Bioscience, Belgium).

### Lipid body staining

Lipid bodies were stained using Bodipy Lipid probe (Thermo Fisher Scientific, USA) as described elsewhere [38]. Cells were harvested and washed twice with PBS containing 5 % ethanol. They were then resuspended in PBS (OD600 = 1), to which 1 % (v/v) Bodipy stock solution (1 mg/mL in ethanol) was added. After 20 min of incubation at room temperature, cells were washed twice with PBS to remove the excess Bodipy. Neutral lipid content was quantified at the single cell level using a BD Accuri™ C6 Flow Cytometer.

### Flow cytometry and data analysis

Lipid bodies stained with Bodipy and cells containing 2-NBDG were detected using a BD Accuri™ C6 Flow Cytometer. For each sample, 40,000 cells were analyzed using the FL1 channel to identify fluorescence associated with Bodipy and 2-NBDG presence (excitation was performed with a 20-mW, 488-nm solid-state blue laser; the emission wavelength was 533/30 nm). Flow rate was set to medium and threshold of 80,000 AU was applied on the forward scatter (FSC) channel for reducing background noise. For both the FL1 and the SSC, the area of the global signal was recorded (FL1-A, SSC-A). The flow cytometry dotplots (FL1/SSC) were analyzed using CFlowPlus software (Accuri, BD Bioscience). For further processing, the raw data were exported as.fcs files and loaded in MatLab using the fca_readfsc function (downloaded from the MatLab File Exchange file server; http://www.mathworks.com). Mean fluorescence was calculated for each sample, and the gates were defined as follows. Gate R1 encompassed 99.9 % of the basal fluorescence signal of the cells (i.e., absence of Bodipy or 2-NDBG). In the hexose transport experiment, gate R3 encompassed intensity values greater than the mean intensity of 2-NDBG fluorescence for cells grown in CMD, while gate R2 encompassed intermediate values (i.e., those between R3 and R1). In the lipid accumulation experiment, gate R2 included intensity values lower than the mean intensity of Bodipy fluorescence for cells grown in CMD, while gate R4 included intensity values higher than the mean intensity of Bodipy fluorescence for cells grown in CMOA. Gate R3 included intermediate values (i.e., those between gates 2 and 4). The percentages represent the percentage of cells found in each gate. Each flow cytometry analysis was performed twice.

## Additional file

10.1186/s12934-016-0558-8 Composition of the medium used in this study.

## Abbreviations

C-mol: carbon molar
Cmol/L: carbon molar per liter
w/w: ratio weight to weight
DM: defined medium
DMD: defined medium glucose
DMG: defined medium glycerol
DMOA: defined medium oleic acid
DMDOA: defined medium glucose oleic acid
DMGOA: defined medium glycerol oleic acid
CM: complex medium
CMD: complex medium glucose
CMG: complex medium glycerol
CMOA: complex medium oleic acid
CMDOA: complex medium glucose oleic acid
CMGOA: complex medium glycerol oleic acid
r_X_: cell growth rate
r_fluo_: specific induction rate
r_G_: specific glycerol uptake rate
r_D_: specific glucose uptake rate
r_OA_: specific oleic acid uptake rate
X_max_: final biomass
gDCW/(L h): gram of dry cell weight per liter and per hour
g/(gDCW h): gram per gram of dry cell weight and per hour
FU: fluorescence unit
FU/h: fluorescence unit per hour
SFU: specific fluorescence unit
PCR: polymerase chain reaction
RT-qPCR: reverse trasnscription quantitative polymerase chain reaction
2-NDBG: 2-(*N*-(7-nitrobenz-2-oxa-1,3-diazol-4-yl)amino)-2-deoxyglucose
WGU: without glucose uptake
HGU: high-glucose-uptake
LLA: low-lipid-accumulation
HLA: high-lipid-accumulation
YPD: yeast extrat peptone dextrose
OD: optical density
YNB: yeast nitrogen base
HPLC: high performance liquid chromatography
PBS: phosphate buffer saline
SSC: side scatter
FSC: forward scatter
nd: not detected
