# Supplementary material for: Deciphering how LIP2 and POX2 promoters can optimally regulate recombinant protein production in the yeast Yarrowia lipolytica
Source: Microb Cell Fact. 2016 Sep 20;15:159. doi: 10.1186/s12934-016-0558-8 (PMC5028966; doi:10.1186/s12934-016-0558-8)
Supplement: Supplementary file 1 — 10.1186/s12934-016-0558-8 Composition of the medium used in this study. [file 12934_2016_558_MOESM1_ESM.docx]

| **Table S1: Composition of the medium used in this study.** | | | | | |
| --- | --- | --- | --- | --- | --- |
|  | CM | DM | Oleic acid | Glucose | Glycerol |
| CMG | + | - | - | - | 1.8 C-mol/L |
| CMD | + | - | - | 1.8 C-mol/L | - |
| CMOA | + | - | 1.8 C-mol/L | - | - |
| CMDOA | + | - | 0.9 C-mol/L | 0.9 C-mol/L | - |
| CMGOA | + | - | 0.9 C-mol/L | - | 0.9 C-mol/L |
| DMG | - | + | - | - | 1.8 C-mol/L |
| DMD | - | + | - | 1.8 C-mol/L | - |
| DMOA | - | + | 1.8 C-mol/L | - | - |
| DMDOA | - | + | 0.9 C-mol/L | 0.9 C-mol/L | - |
| DMGOA | - | + | 0.9 C-mol/L | - | 0.9 C-mol/L |
| - CM basis is composed as follows: 0.93 g/L CaSO_4_.2H2O, 18.2 g/L K_2_SO_4_, 7.28 g/L MgSO_4_.7H_2_O, 4.4 g/L KOH, 26.7 mL/L H_3_PO_4_ 85 %, 10 mg/L FeCl_3_, 1 g/L glutamate, 5 mL/L of PTM1 solution (6 g/L CuSO_4_.5H_2_O, 0.08 g/L KI, 3 g/L MnSO_4_.2H_2_O, 0.2 g/L Na_2_MoO_4_.2H_2_O, 0.02 g/L H_3_BO_3_, 0.5 g/L CoCl_2_.6H_2_O, 20 g/L ZnCl_2_, 6.5 g/L FeSO_4_.7H_2_O and 5 mL/L H_2_SO_4_) as well as 2 mL/L of a vitamin solution (8 μg/L biotin, 200 μg/L thiamin 4 μg/L myo-inositol.  - DM basis is composed of 10 g/L yeast extract and 20 g/L tryptone.  - The + sign indicates the presence of the compound or medium basis (CM or DM) while the sign – indicate its absence. | | | | | |
